# Poor Law Topics

**Published:** 1906-02-24

**Authors:** 


### POOR LAW TOPICS.

### MR. BURNS AND LABOUR COLONIES.

Practical people will rejoice that one of Mr. Burns's first actions as President of the Local Government Board has been to refuse consent to the application of the Lambeth Guardians for powers to expend £12,000 on the purchase of land for a labour colony. Mr. Burns is himself no believer in labour colonies as a panacea for the evils of unemployment, and his opinions on the problems of labour and the labouring class are worth considering. But, apart from his personal views, there is certainly sound sense in the reasons given for refusing to accede to Lambeth's request. Labour colonies are as yet in an experimental condition. It is too soon yet for anyone to say definitely that they keep the temporarily unemployed in physical and moral efficiency. Some people believe that they do, and if these people have the will and the power to back their opinion with money they are justified in doing so. We can gratefully commend Mr. Fels's action as showing a genuine philanthropic impulse, and all will unite in hoping that his experiments may be successful. But, until this is proved, it is not right that money drawn compulsorily from the public should be devoted to an object of which the success is still doubtful. Labour colonies are not a cheap way of dealing with the unemployed. It is reckoned that the cost of maintaining a man at a colony and his family in town averages 30s. a week. The work he does is very rarely sufficient to cover his maintenance. This is no reproach to the labourer, for it is as a rule work entirely different from what he is accustomed to; but it implies that, as work which is to fit him for that return to the land which many people desire, it is still a failure. Left to himself, he would probably be unable to earn a living at any country job; and, as a matter of fact, very few of the men wish to remain in the country. So far, therefore, there is no proof that a labour colony has any educational or remedial efficiency. There remains the question of the maintenance of physical strength. But a workhouse ought not to be—and, as a matter of fact, is not—an abode of dreary idleness. In many of them a skilled workman has a good chance of being set to work at his trade, and rougher work can as a rule be found for the unskilled. Some unions have this winter given outdoor relief to the families of unemployed men on condition that the men themselves go into the workhouse. This seems to carry out the underlying principle of the labour colony without a great capital expenditure which, if trade improves, might be absolutely useless in the future. The Lambeth ratepayers at least should be grateful to the President of the Local Government Board for refusing consent to an immediate expenditure of £12,000 for land, with the prospect of further expenditure of unknown amount, with a very doubtful prospect of permanent advantage.

### THE INSURANCE OF PAUPERS.

Does the money for which a man's life is insured form part of his estate? This question is certain to be raised before long. Recently a man died whose life was insured seven times in favour of as many different persons. Yet none of these would pay the expenses of his funeral, and the parish authorities had to bury him. An incident like this seems so grossly unjust that it is doubtful if the authorities will endure it in silence. In most cases an insurance policy is regarded as part of a man's personalty, and death-duty is paid on it accordingly. Certainly it could be drawn upon for the payment of his debts, among which funeral expenses would be counted. That a man should insure his life for the benefit of his family is quite right, but the family are bound to use the money in the first place for the payment of debts. Possibly the insurances in the case in question were taken out by others for their own benefit, and the premiums paid by them. But this is always a proceeding of doubtful fitness, and in good companies the question of an "insurable interest" in the life insured is always insisted on—that is, such an interest as justifies the company in believing that the insurer has more reason to desire the life than the death of the person insured. If such an interest does not exist, the whole thing becomes a sort of gruesome gamble with death, which should not be encouraged. That such insurances often lead to crime is well known. There seems to have been no such sin committed in the case in question, but the view of seven harpies waiting for a man to die is revolting. That they had little of either affection or respect for the dead man is clearly shown by their refusal to expend any part of their profits in showing him that last respect to render which is so much a point of honour with the decent poor.

### THE FIREWOOD QUESTION.

The "Firewood Trades Association," whose existence will be a surprise to most people, have protested against the Paddington Guardians setting the inmates of their workhouse to chopping wood and making it up into bundles of firewood for sale. The Guardians have replied, sensibly, that they must occupy the inmates in some way. In all workhouses as many inmates as possible are employed in the work of the place, and in some institutions a great many necessaries—boots, clothing, tinware—are made by inmates. We have seen an old bookbinder cheerfully employed at his former trade, making up into volumes the magazines which good-natured people sent to the paupers. But all this implies some skill on the part of the worker, and a certain permanence of residence in the workhouse. It is a very good thing to set the decent old people to some such work, and reward them by a little extra luxury. But especially in the winter the workhouses are filled with unskilled and often disreputable able-bodied paupers. They cannot be set to any of the regular tasks of the place; there is not work enough to go round, and they could not be trusted with it if there was. Are they therefore to lounge about in idleness until with the return of spring they incline to go out into the world again? Looking round for something for these people to do, Guardians find themselves with but little choice. The general selection is stone-breaking for heavy work and wood-chopping for the less able. The wood being chopped, what is to be done with it? If it cannot be used in the institution it must be sold, and surely all that the Firewood Association can ask is that it shall not be sold at less than the ordinary price. If there had been underselling their protest would have been justifiable, but if the ordinary price is maintained there is no more than ordinary fair competition. The Church Army and the Salvation Army do wood-chopping also, and for the same reason—that it is better, even when a man is being kept by charity, that he should do something for his maintenance. But the result of that work when produced must be disposed of, or the demands on the charitable public would reach a point that could not be met. The same holds good of paupers. It is better for them to work, and it is wholesome also that they should feel that their work has a market value. If the Paddington Guardians had accepted as reasonable the protest of the Firewood Trades' Association, they would have been pestered with the protests of every trade that is represented in the workhouse, until it would have been an offence to set a pauper to put a coat of paint on a wall or patch a coat. And "workhouse" would have been a misnomer for the place.

